# Postoperative exogenous endophthalmitis caused by *Escherichia coli:* a rare case report and literature review

**DOI:** 10.2144/fsoa-2022-0076

**Published:** 2023-04-04

**Authors:** Rami A Al-Dwairi, Abdelwahab Aleshawi, Zaki Shannak, Wafa Al-Shorman, Seren Al Beiruti, Ahmed Al Sharie

**Affiliations:** 1 Department of Special Surgery, Division of Ophthalmology, Faculty of Medicine, Jordan University of Science & Technology, Irbid, 22110, Jordan

**Keywords:** endogenous, endophthalmitis, *Escherichia coli*, exogenous

## Abstract

**Aim:**

We report a rare case of postoperative endophthalmitis caused by *Escherichia coli*.

**Case description:**

The diagnosis of postoperative endophthalmitis in our patient was established based on the clinical signs of hypopyon along with vitritis. The patient underwent pars plana vitrectomy, anterior chamber washout, intraocular lens removal, and intravitreal antibiotics (amikacin and vancomycin) injection. The culture of both the vitreous sample and the intraocular lens, revealed a heavy growth of *Escherichia coli*.

**Conclusion:**

*Escherichia coli* is an unusual microorganism to cause postoperative endophthalmitis. A major breach in the sterilization may explain this infection. Proper sterilization and prophylactic measures are crucial to avoid this disastrous complication.

## Plain language summary

Endophthalmitis is a rare catastrophic sight threatening complication after cataract surgery. It is commonly caused by Gram–positive bacteria. In this rare case report, we present a case of postoperative cataract surgery caused by *Escherichia coli*; a Gram-negative bacteria. Proper prophylactic measures in the operation rooms along with proper prophylactic antibiotics may aid in avoiding such complications by such rare microorganism.

Endophthalmitis is by far one of the most catastrophic sight threatening diseases in ophthalmology. It is defined as an inflammation, almost always secondary to infection, affecting either the intraocular tissues or fluids (usually including the vitreous) [1,2]. Endophthalmitis are categorized into exogenous or endogenous, depending on the pathway by which the causative organism was introduced into the eye [3,4]. Endogenous endophthalmitis, which is less common than exogenous endophthalmitis (accounting for only 2–6% of all cases of endophthalmitis) comes from a hematogenous dissemination of microorganism from a distant source in the body, with both bacterial and fungal causes predominating this category [5–7].

On the other hand, exogenous endophthalmitis is caused by an intraocular inoculation of a microorganism of an external origin; any breach to the eye can introduce such microorganisms. Trauma is the most common cause, since it has been reported that between 3 and 17% of eyes after penetrating injuries present intraocular infection [8]. Exogenous endophthalmitis can present also following intraocular surgery, with an incidence currently ranging from 0.03 to 0.15% worldwide. In recently published series, between 49.6 and 66.9% of cases of endophthalmitis were secondary to trauma, and between 26.7 and 29.5% presented after intraocular surgery. Exogenous postoperative endophthalmitis can in turn have an endogenous or an exogenous source, depending on whether the microorganisms came from the flora of the same patient, or they were originated in an external source [9–11]. Staphylococci and streptococci are the most common causative agents of postoperative endophthalmitis and they normally reside on eyelid margins and pre-ocular tear film, hence the less virulent course compared with post-traumatic endophthalmitis, which is caused by microorganisms coming from the environment (such as bacillus species and fungi) [12].

In this case report we present a postoperative exogenous endophthalmitis caused by *Escherichia coli* which has been seldom documented in the current literature.

## Case description

A 50-year-old female, not known to have any medical illnesses, underwent phacoemulsification with intraocular lens implant in her right eye, elsewhere, presented to our ophthalmic emergency department 6 days after the surgery complaining of severe right eye pain for 2 days. The patient was using topical eye drops containing antibiotic (moxifloxacin) and steroids (prednisolone acetate) in right eye. The patient had glaucoma, and she was on dorzolamide, timolol and latanoprost in both eyes.

On examination, the visual acuity in right eye was light perception, anterior segment exam revealed hypopyon 1.5 mm and dense fibrinous reaction along with posterior synechia and ciliary injection. There was no view for the posterior segment on the dilated fundus exam. B scan ultrasonography was done and revealed vitreitis. The intraocular pressure (IOP) was 6 mmHg.

**Figure 1.**
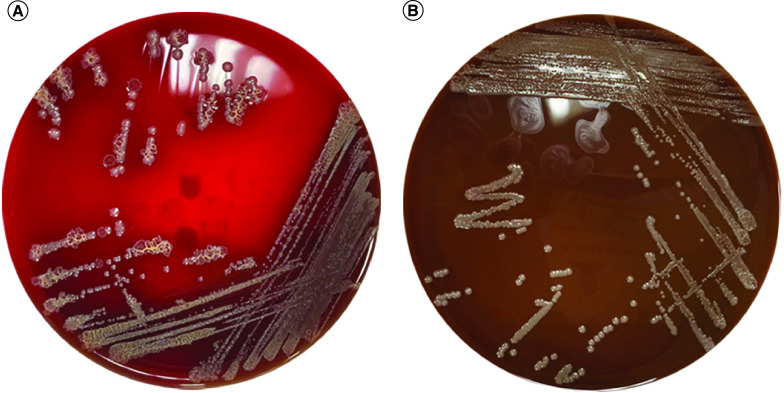
Culture of the vitreal sample. **(A)** Blood agar and **(B)** chocolate agar showed heavy growth of *Escherichia coli*.

Diagnosis of acute postoperative endophthalmitis was established. Accordingly, the patient was admitted and started on empirical intravenous antibiotics. Pars plana vitrectomy, anterior chamber washout, intraocular lens removal (IOL), and intravitreal vancomycin and amikacin injections were done on the same day. During vitrectomy, removal of dense fibrin at the vitreous base was performed and the disc was found to be subtotally cupped. An iatrogenic tear with focal detachment inferiorly noticed so aspiration of subretinal fluid and endolaser was done. The retina kept flat under sulfur hexafluoride (SF6) gas. A vitreal sample and the extracted IOL were sent for microbiology culture and sensitivity test and both revealed a heavy growth of *Escherichia coli*, Figure 1. The *Escherichia coli* was sensitive to amikacin.

**Figure 2.**
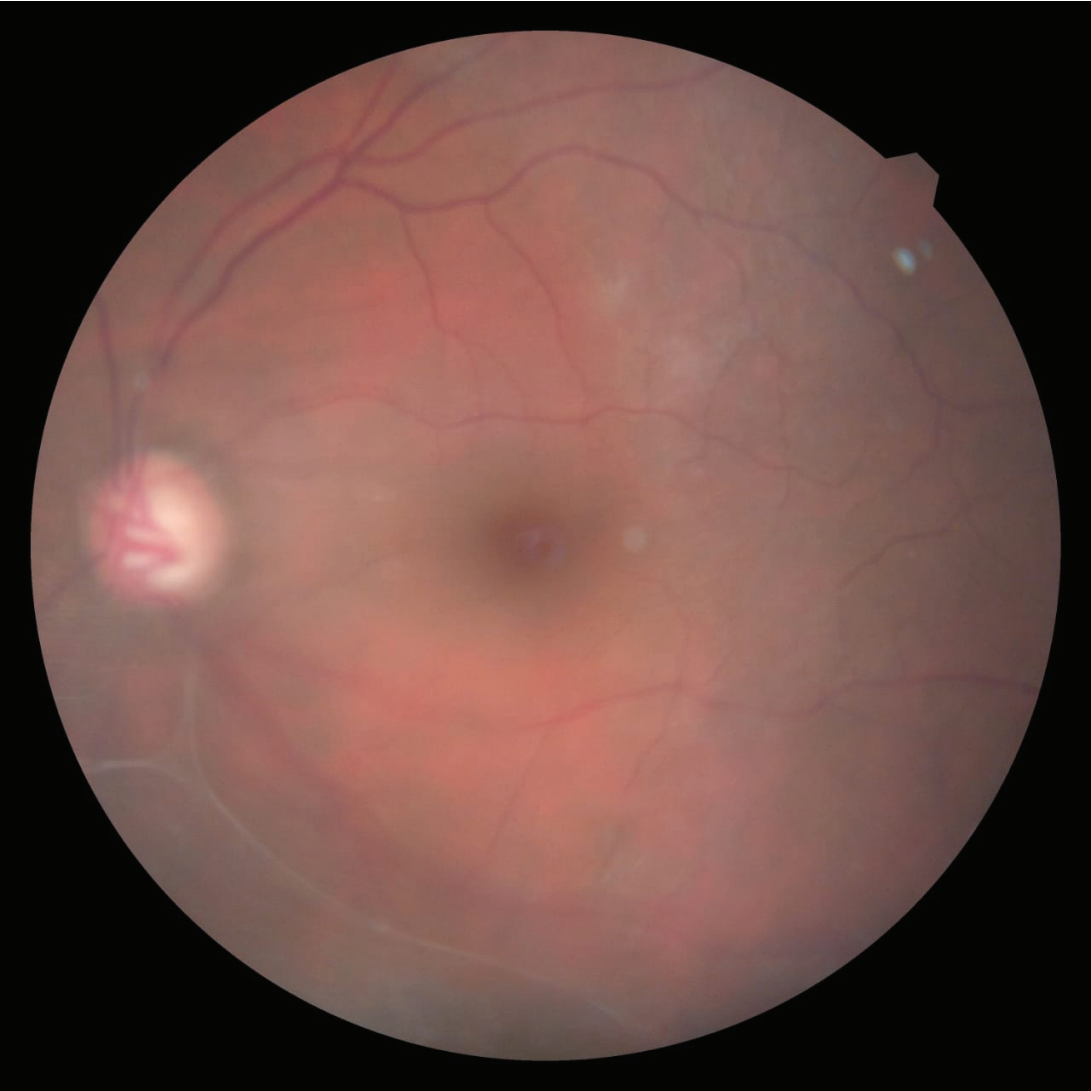
Fundus photo showed subtotal cupping of the optic disc due to glaucoma.

Postoperative day 1, the vision was light perception, anterior segment showed corneal edema +1, descemet folds +1 and deep anterior chamber. There was no view of the posterior segment and IOP was 14. She was discharged on fortified antibiotics amikacin and vancomycin, prednisolone acetate and her antiglaucoma medications. One week later, her vision was improved to hand motion, vitreous chamber showed gas level of 20% and intraocular pressure of 10 mmHg. On the last follow-up visit (6 months postoperative), the cornea was transparent, and the macula looked normal, but the visual acuity was hand motion, which may be probably explained mainly by the subtotally cupped disc, Figure 2.

## Discussion

To the best of our knowledge, we report the second case *Escherichia coli*-postoperative endophthalmitis. Kim *et al.* reported a case of post phacoemulsification endophthalmitis caused by extended-spectrum Beta-lactamase-producing *Escherichia coli* that did not respond to multiple types of intravitreal antibiotics, and the patient finally had no light perception visual acuity [13]. Regarding post intravitreal injection endophthalmitis, only one case of *Escherichia coli* was reported by Irigoyen *et al.* [14].

Endophthalmitis is a sight-threatening ophthalmic emergency that is defined by the inflammation of the internal ocular space, which in majority of the cases has an infective source. Postoperative endophthalmitis remains the most feared complication by ophthalmologists. As we are going into the era of a more aging population worldwide, the number of cataract surgeries performed is increasing, rendering post-cataract surgery endophthalmitis a more common concern [15].

Visual outcomes of this complication are not often favorable, where only 33 % of cases reach visual acuity of better than 20/40 and about 40% of affected patients end up with severe visual loss (corrected distance visual acuity of less than 20/200), and some may even end up in evisceration leading to high morbidity [16]. Knowing the common causative microorganism of this morbid complication of cataract surgery is important as it helps to tailor the appropriate prophylaxis, in which numerous fungal and bacterial agents can cause post-cataract surgery endophthalmitis, with Gram–positive, coagulase-negative staphylococci and streptococci being the most common as these microorganisms generally reside on the eyelid margins and pre-ocular tear film. However, Gram-negative bacteria (which show a higher degree of virulence as compared with Gram-positive ones) may also be encountered occasionally, in which the most reported pathogen is *Klebsiella pneumonia* [12]. The Gram-negative bacteria, as *Escherichia coli* are more commonly to cause endogenous endophthalmitis or post-traumatic endophthalmitis [17–20].

Many studies recommended the use of intracameral antibiotics as a prophylactic measure against endophthalmitis. The ESCRS study suggested intracameral cefuroxime (1.0 mg in 0.1 ml) at the time of cataract surgery for prevention of postoperative endophthalmitis. Cefuroxime is a second-generation cephalosporin that is sensitive to Gram-positive cocci [21]. In another multicenter study, Haripriya *et al.* used intracameral moxifloxacin (0.5 mg in 0.1 ml) and showed threefold (for manual small incision cataract surgery) to sixfold (for phacoemulsification) reduction in the occurrence of endophthalmitis. Moxifloxacin is a fourth-generation fluoroquinolone that is sensitive to Gram-positive cocci including MRSA and selective Gram-negative bacilli [22].

The main limitation point is the lack of information regarding the details of the primary cataract surgery and any possibility regarding any breakdown that may cause this complication.

## Conclusion

Postoperative endophthalmitis caused by *Escherichia coli* is a rare complication. A major breach in the pathway of the surgical system may result in this catastrophic complication. Proper sterilization and prophylactic measures should be carried out.

**Summary points**

- Endophthalmitis is a rare disastrous sight threatening complication after cataract surgery.
- Gram–positive staphylococci and streptococci being the most common as these microorganisms to cause postoperative endophthalmitis.
- Gram-negative bacteria uncommonly cause postoperative endophthalmitis.
- We report the second case of postoperative endophthalmitis caused by *Escherichia coli*.
- Proper prophylactic measures and antibiotics should be carried out properly.
